# Laugier–Hunziker Syndrome in an 8-Year-Old Boy with Scleral Melanocytosis, Lingual Pigmentation, Labial Pigmentation, and Melanonychia Striata

**DOI:** 10.1155/2020/8267805

**Published:** 2020-03-17

**Authors:** Alexander K. C. Leung, Kin Fon Leong, Benjamin Barankin, Joseph M. Lam

**Affiliations:** ^1^The University of Calgary, Calgary, Canada; ^2^The Alberta Children's Hospital, Calgary, Alberta T2M 0H5, Canada; ^3^Pediatric Institute, Kuala Lumpur General Hospital, Kuala Lumpur, Malaysia; ^4^Toronto Dermatology Centre, Toronto, Ontario M3H 5Y8, Canada; ^5^Department of Dermatology and Skin Sciences, University of British Columbia, Vancouver, British Columbia V5Z 1K1, Canada; ^6^BC Children's Hospital, Vancouver, British Columbia, Canada

## Abstract

Laugier–Hunziker syndrome is a rare, acquired disorder characterized by mucocutaneous hyperpigmentation and melanonychia striata with no underlying systemic abnormalities. We report an 8-year-old boy with Laugier–Hunziker syndrome who presented with melanonychia striata affecting all the fingernails and toenails, macular pigmentation on the tongue and the lower lip, and scleral melanocytosis. Melanonychia striata rarely affect all the twenty nails, and scleral melanocytosis has rarely been reported in association with Laugier–Hunziker syndrome. Laugier–Hunziker syndrome occurs predominately in adults. Our patient is the youngest reported patient with Laugier–Hunziker syndrome.

## 1. Introduction

Laugier–Hunziker syndrome, also known as Laugier–Hunziker–Baran syndrome, Laugier–Gerbig–Hunziker syndrome, or idiopathic lenticular mucocutaneous pigmentation, is a rare, acquired disorder characterized by mucocutaneous hyperpigmentation and melanonychia striata with no underlying systemic abnormalities [1]. The condition was first described in 1970 by Laugier and Hunziker who reported five patients with acquired macular hyperpigmentation of the lips and oral mucosa and two of these patients displayed hyperpigmentation of the fingernails [2]. Since the description of the disorder, approximately 200 cases have been reported [3, 4]. The majority of cases have been reported in adults. Herein, we describe an 8-year-old Bajau boy with Laugier–Hunziker syndrome who presented with melanonychia striata affecting all the fingernails and toenails, macular pigmentation on his tongue and lower lip, and scleral melanocytosis. Melanonychia striata rarely affect all the twenty nails, and scleral melanocytosis has rarely been reported in association with Laugier–Hunziker syndrome [5]. To our knowledge, our patient is the youngest reported patient with Laugier–Hunziker syndrome.

## 2. Case Report

An otherwise healthy 8-year-old Bajau boy from Malaysia presented with a history of longitudinal and diffuse melanonychia over all his nails which had developed gradually over the last two years and increasing macular pigmentation on his tongue, lower lip, and right eye which had developed gradually over the last year. The lesions were asymptomatic. Functional enquiry was negative. In particular, there was no history of malaise, fatigue, fainting episodes, weight loss, recurrent abdominal pain, intermittent vomiting, gastrointestinal bleeding, palpitation, or shortness of breath. There was no relevant drug history. Parents were nonconsanguineous. There was no family history of mucocutaneous pigmentary disorders or intestinal polyposis. The child had been seen eight months previously by a pediatric gastroenterologist who made a provisional diagnosis of Peutz–Jeghers syndrome. Subsequently, the child underwent an esophagogastroduodenoscopy and colonoscopy with normal results.

**Figure 1:**
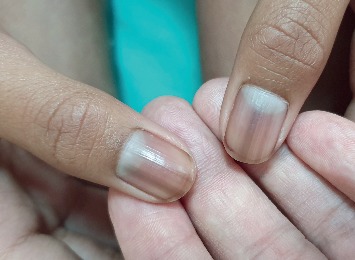
Longitudinal and homogeneous pigmentation on the patient's fingernails.

**Figure 2:**
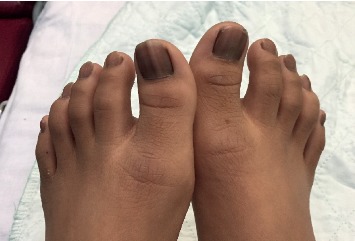
Longitudinal and homogeneous pigmentation on the patient's toenails.

**Figure 3:**
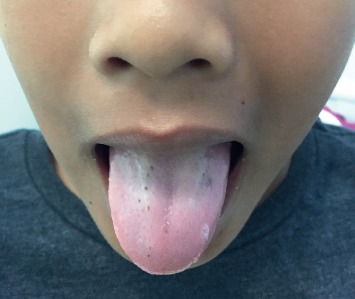
Discrete brown black macules on the tongue.

**Figure 4:**
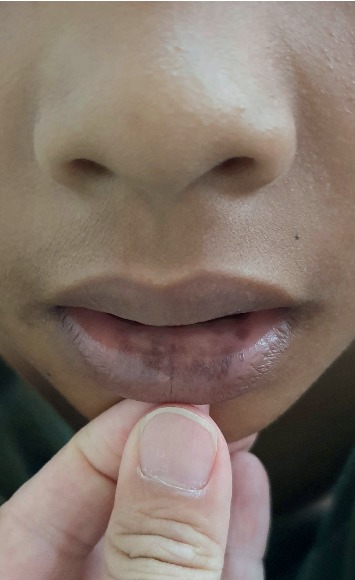
Discrete brown black macules on the lower lip.

On physical examination, the child had melanonychia striata affecting all the fingernails (Figure 1) and toenails (Figure 2) with no nail dystrophy, discrete brown-black macules on the tongue (Figure 3) and the lower lip (Figure 4), and a well-defined pigmented macule on the nasal aspect of the right sclera. The rest of the physical examination was unremarkable.

Complete blood cell count, serum electrolytes, serum cortisol, serum adrenocorticotropic hormone, liver function tests, chest radiograph, ultrasound of the abdomen, electrocardiogram, and echocardiography were normal. Given the pigmented macules on the tongue as well as the lower lip and melanonychia striata, the absence of somatic abnormalities, negative history of medication intake, and normal laboratory investigations, a diagnosis of Laugier–Hunziker syndrome was made. The parents were reassured of the benign nature of the disorder and that treatment was not necessary. Our patient and his parents did not have any cosmetic concerns. As such, no treatment was given.

## 3. Discussion

Laugier–Hunziker syndrome is an acquired disorder which occurs predominately in adults with a mean age of onset of 50 years [6]. Occurrence before puberty is uncommon [6]. Thus far, the youngest child reported to have Laugier–Hunziker syndrome was a 12-year-old boy who presented with perioral, intraoral, and palmar pigmentation [7]. Herein, we report an 8-year-old boy with Laugier–Hunziker syndrome.

Laugier–Hunziker syndrome, a benign disease with no systemic manifestations, is characterized by asymptomatic mucocutaneous hyperpigmentation and melanonychia striata with no underlying systemic abnormalities [1]. Mucosal involvement in Laugier–Hunziker syndrome is characterized by asymptomatic, gray, light brown, brown, brown-black, blue-black, or black macules on the oral and genital mucosa [1, 3, 6]. The macules usually have well-defined margins which may be oval, lenticular, linear, or irregular in shape and less than 5 mm in diameter [3, 6, 8, 9]. These macules may be solitary or multiple and sometimes confluent [9]. Oral lesions are commonly seen on the labial mucosa (particularly the lower lip) and buccal mucosa [6, 8, 9]. Less frequently, the lesions are present on the gingiva, palate, floor of the mouth, and tongue [6, 8, 9]. Rarely, the lesions are on the conjunctiva [10–12]. Mucosal lesions in the genitalia are seen in the vulva and/or vagina in females and glans penis in males [1, 8].

Ocular pigmentation is rare. A perusal of the literature revealed seven cases of hyperpigmented conjunctival macules [8, 10–14] and only one case of scleral melanocytosis in the context of Laugier–Hunziker syndrome [15]. In 2000, Began and Mirowski reported a 67-year-old African American with Laugier–Hunziker syndrome presenting with pigmented macules on the hard palate, buccal mucosa, gingiva, inner vermillion border of both lips, palms, and soles as well as a brown-black macule on the right sclera [15]. The present case adds another example of scleral melanocytosis in association with Laugier–Hunziker syndrome. Scleral melanocytosis, a congenital melanocytic hyperpigmentation of the sclera, is a common racial characteristics of Asian and black subjects [16, 17]. Leung et al. examined 2914 Chinese children (1510 males and 1404 females) for the presence of scleral melanocytosis in a cross-sectional prevalence survey [17]. Scleral melanocytosis was found in 4.9% of boys and 4.1% of girls under one year of age. The peak prevalence was at 6 years of age when 44.6% of boys and 46.6% of girls were found to have scleral melanocytosis. The prevalence decreased thereafter. At 18 years of age, only 11.1% of boys and 13.2% of girls were found to have scleral melanocytosis. We are not sure whether the occurrence of scleral melanocytosis in these two patients with Laugier–Hunziker syndrome is coincidental or causally related. It is likely that the occurrence of ocular hyperpigmentation in Laugier–Hunziker syndrome might be more common than is generally appreciated due to under-reporting of cases. It is hoped that similar cases will be forthcoming to firm up the association. In the mean time, we suggest ocular pigmentation be grouped under the umbrella “essential melanotic pigmentation of mucosa.”

Melanonychia striata is present in approximately 50 to 60% of patients with Laugier–Hunziker syndrome and may present as single, double, or multiple longitudinal stripes that runs along the longitudinal axis of the nail from the proximal nail fold to the distal nail plate or, less commonly, as homogeneous pigmentation involving one half of the nail or complete nail [3, 6, 9, 18]. The coloration can be tan, brown, gray, or black [18]. Fingernails are affected more often than toenails [3, 5]. Melanonychia striata is more common in the thumb. Involvement of multiple digits is less common and is most commonly seen in dark-skinned individuals and individuals with connective tissue disorders [5, 18, 19]. Our case is unique that all the fingernails and toenails were affected. To the best of our knowledge, melanonychia striata involving all twenty nails has not been reported either in the general population or in patients with Laugier–Hunziker syndrome [5].

Laugier–Hunziker syndrome is a rare disease and diagnosis can be challenging. Differential diagnosis includes physiologic (racial) pigmentation seen in dark-skinned individuals (pigmentation most commonly seen in the gingiva, tips of the fungiform papillae on the dorsal surface of the tongue, and nails as melanonychia striata), Peutz–Jeghers syndrome (autosomal dominant mode of inheritance, intestinal polyposis, mucocutaneous melanotic macules present at birth or in very early life, labial pigmentation may cross the vermilion border, absence of nail involvement, and presence of *STK11* gene mutation), Addison disease (fatigability, lethargy, nausea, vomiting, weight loss, salt craving, weight loss, hypotension, hyperpigmentation of the skin, palmar creases, knuckles, and mucosa, hypoglycemia, hyponatremia, hyperkalemia, and low serum cortisol with elevated adrenocorticotropic hormone), LEOPARD syndrome (Lentigines, Electrocardiographic conduction defects, Ocular hypertelorism, Pulmonary stenosis, Abnormalities of the genitalia, Retarded growth, and Deafness), Carney complex also known as LAMB syndrome (Lentigines, Atrial myxomas, Mucocutaneous myxomas, and Blue nevi), Bandler syndrome (hyperpigmented macules on the hands, oral mucosa, and nails and intestinal vascular malformation), Cronkhite–Canada syndrome (hyperpigmentation with more proximal involvement, diffuse gastrointestinal polyposis, alopecia, and onychodystrophy), neurofibromatosis type 1 (café au lait macules, cutaneous neurofibromas, subcutaneous neurofibromas, plexiform neurofibromas, intertriginous freckling, optic glioma, iris Lisch nodules, and musculoskeletal abnormalities), McCune–Albright syndrome (café au lait macules with irregular border simulating “coast of Maine” often with unilateral truncal distribution, polyostotic fibrous dysplasia, and hyperfunctioning endocrinopathies including precocious puberty and no nail involvement), and medication-induced pigmentation (minocycline, oral contraceptives, amiodarone, levodopa, ketoconazole, clofazimine, zidovudine, phenothiazine, chlorpromazine, hydroxyurea, cyclophosphamide, doxorubicin, bleomycin, chloroquine, and hydroxychloroquine) [1, 4, 6, 20].

Laugier–Hunziker syndrome should be suspected in the presence of melanonychia striata and mucocutaneous pigmentation with onset of mucosal lentigines later in life rather than at birth or shortly thereafter. The lack of systemic symptoms (e.g., fatigue and weight loss), dysmorphic features, gastrointestinal disorders (e.g., vomiting, abdominal pain, and gastrointestinal bleeding), cardiovascular disorders (e.g., dizziness, shortness of breath, orthopnea, chest tightness, angina, cold sweats, arrhythmia, and ankle edema), history of medication intake, normal plasma levels of cortisol and adrenocorticotropic hormone, and negative findings in chest radiograph, ultrasound of the abdomen, gastroduodenoscopy, colonoscopy, electrocardiogram, and echocardiography support the diagnosis of Laugier–Hunziker syndrome. This case fits most into Laugier–Hunziker syndrome given the late onset of mucosal lentigines rather than at birth or shortly thereafter, presence of melanonychia striata, absence of gastrointestinal symptoms and intestinal polyps, and negative family history.

## 4. Conclusion

Laugier–Hunziker syndrome occurs predominately in adults, and the mean age of diagnosis is 50 years. Our patient is the youngest child reported to have Laugier–Hunziker syndrome. Other interesting features of our case include pigmented macules on the tongue and the lower lip, sparing of acral area and palmoplantar area, melanonychia striata involving all the fingernails and toenails, and the presence of scleral melanocytosis which prompted the present presentation.
